# Molecular differences between lymph nodes and distant metastases compared with primaries in colorectal cancer patients

**DOI:** 10.1038/s41698-021-00230-y

**Published:** 2021-10-27

**Authors:** Alberto Puccini, Andreas Seeber, Joanne Xiu, Richard M. Goldberg, Davide Soldato, Axel Grothey, Anthony F. Shields, Mohamed E. Salem, Francesca Battaglin, Martin D. Berger, Wafik S. El-Deiry, Ryuma Tokunaga, Madiha Naseem, Wu Zhang, Sukeshi Patel Arora, Moh’d M. Khushman, Michael J. Hall, Philip A. Philip, John L. Marshall, W. Michael Korn, Heinz-Josef Lenz

**Affiliations:** 1 Norris Comprehensive Cancer Center, Keck School of Medicine, University of Southern California, Los Angeles, CA USA; 2 University of Genoa, Medical Oncology Unit 1, Ospedale Policlinico San Martino, Genoa, Italy; 3 Department of Hematology and Oncology, Comprehensive Cancer Center Innsbruck, Medical University of Innsbruck, Innsbruck, Austria; 4 Caris Life Sciences, Phoenix, AZ USA; 5 West Virginia University Cancer Institute, Morgantown, WV USA; 6 West Cancer Center, University of Tennessee, Germantown, TN USA; 7 Department of Oncology, Karmanos Cancer Institute, Wayne State University, Detroit, MI USA; 8 Levine Cancer Institute, Carolinas HealthCare System, Charlotte, NC USA; 9 Brown University and Lifespan Cancer Institute (LCI), Providence, RI USA; 10 Mays Cancer Center, UT Health San Antonio San Antonio, San Antonio, TX USA; 11 The University of South Alabama, Mitchell Cancer Institute, Mobile, AL USA; 12 Medical Oncology and Population Sciences, Fox Chase Cancer Center, Phoenix, AZ USA; 13 Ruesch Center for The Cure of Gastrointestinal Cancers, Lombardi Comprehensive Cancer Center, Georgetown University Medical Center, Washington, DC USA

**Keywords:** Cancer genomics, Colon cancer

## Abstract

Lymph nodes (LNs) and distant metastases can arise from independent subclones of the primary tumor. Herein, we characterized the molecular landscape and the differences between LNs, distant metastases and primary colorectal cancers (CRCs). Samples were analyzed using next generation sequencing (NGS, MiSeq on 47 genes, NextSeq on 592 genes) and immunohistochemistry. Tumor mutational burden (TMB) was calculated based on somatic nonsynonymous missense mutations, and microsatellite instability (MSI) was evaluated by NGS of known MSI loci. In total, 11,871 samples were examined, comprising primaries (*N* = 5862), distant (*N* = 5605) and LNs metastases (*N* = 404). The most frequently mutated genes in LNs were *TP53* (72%), *APC* (61%), *KRAS* (39%), *ARID1A* (20%), *PIK3CA* (12%). LNs showed a higher mean TMB (13 mut/MB) vs distant metastases (9 mut/MB, *p* < 0.0001). TMB-high (≥17mut/MB) and MSI-H (8.8% and 6.9% vs 3.7%, *p* < 0.001 and *p* = 0.017, respectively) classifications were more frequent in primaries and LNs vs distant metastases (9.5% and 8.8% vs 4.2%, *p* < 0.001 and *p* = 0.001, respectively). TMB-high is significantly more common in LNs vs distant metastases and primaries (*P* < 0.0001), regardless MSI-H status. Overall, LNs showed significantly different rates of mutations in *APC, KRAS, PI3KCA, KDM6A*, and *BRIP1* (*p* < 0.01) vs primaries, while presenting a distinct molecular profile compared to distant metastases. Our cohort of 30 paired samples confirmed the molecular heterogeneity between primaries, LNs, and distant metastases. Our data support the hypothesis that lymphatic and distant metastases harbor different mutational landscape. Our findings are hypothesis generating and need to be examined in prospective studies.

## Introduction

Colorectal cancer (CRC) is a highly heterogeneous disease caused by multiple genetic and epigenetic triggers^1,2^. Spreading of cancer cells from primary colorectal tumors to regional lymph nodes and to distant organs is associated with reduced survival. While stage 1 disease the 5-year overall survival (OS) approaches 90%, in the metastatic stage the 5-year OS rate decreases to approximately 10–15%^3^. One hypothesis to explain this association proposes that distant metastases are seeded by lymph nodes metastases. This view provides a mechanistic basis for the TNM staging system and is often the rationale for surgical resection of tumor-draining lymph nodes. However, it is still unclear whether a single metastatic subclone evolves in the primary tumor, subsequently spreading to lymph nodes and distant sites, or whether multiple subclones in the primary tumor become dispersed and independently seed lymph nodes and distant metastases.

A study published by Naxerova and colleagues takes the issue one important step further^4^. They have modeled a phylogenetic tree of subclones spreading to lymph nodes and/or distant organs using in -depth evaluation of hypermutable DNA regions. In the vast majority of cases, they could show that independent subclones are responsible for lymphatic and hematogenic spreading. This model is consistent with previous studies demonstrating that somatic exonic mutations in a colon cancer patient’s liver metastases are not only identical to those found in the patient’s lymph nodes deposits but are also found in samples from the primary tumor site^5^. Interestingly, it has been shown by the The Cancer Genome Atlas (TCGA) consortium that exon mutations do not differ between colon cancers that do and those that do not metastasize^1,6^.

To date, only a few studies have investigated the molecular profile and the mutational heterogeneity of lymph nodes metastases. Zhang and colleagues showed that skip spreading of tumor cells within the lymphatic network occurs frequently^7^. Furthermore, Ulintz et al.^8^, only recently, found that lymph nodes metastases are polyclonal and differ considerably from one lymph node to another. Additionally, they could show that a single lymph node can harbor subclones that arise in different geographic regions of the primary tumor. These findings are consistent with a model of metastasis where multiple waves of metastatic cells escape the primary tumor over time and seed lymph nodes and other metastatic sites during tumor progression^8^.

Recent population-based studies on metastases in CRC showed that between 40%^9^ and 63%^10^ of metachronous metastases develop in patients without lymph nodes metastases, demonstrating that distant metastases can develop independently of the presence of lymph nodes metastases^11^. The possibility to individuate those clones with a predilection to seed lymph nodes versus those with a proclivity to cause distant metastases could have an impact on clinical practice, especially for early- stage CRC patients. In fact, it is well-known that stage III CRCs are heterogeneous in their clinical behavior^12^. Although there is insufficient evidence for changing the diagnostic workup or treatment of CRC or the TNM staging system based solely on the findings of the study from Naxerova et al.^4^, this study provokes innovative thinking about its implications to shape future translational research and improve treatment. In addition, Hu Z. and colleagues showed that the genomic divergence between the primary tumor and metastasis is low and that canonical driver genes are acquired early in CRC patients with liver and brain metastasis^13^. They also observed that early in the natural history of tumors, 81% of the time disseminating cells seed metastases even before a primary carcinoma is clinically detectable.

More extensive molecular characterization of the primary tumor and metastases might provide further clues in this respect. Therefore, with this study we aimed to describe the molecular features of lymph node metastases deriving from CRC. Subsequently, we have compared lymph nodes and distant metastases to evaluate whether molecular differences exist between these metastatic sites and primary tumors.

## Results

### Patient demographics

**Fig. 1:**
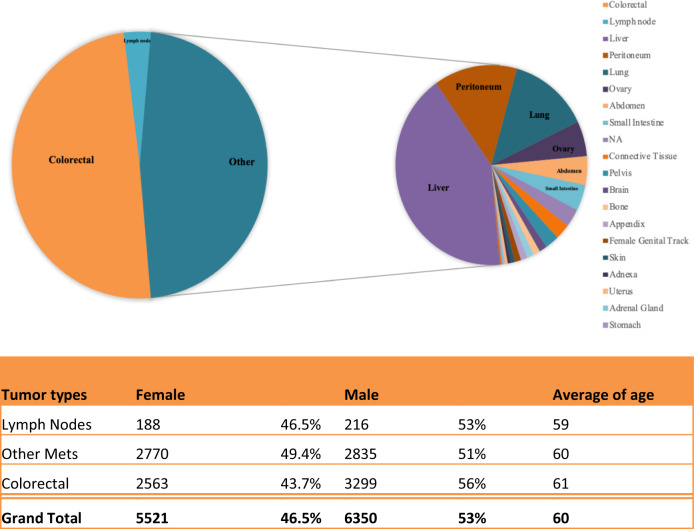
Patients demographics.

A total of 11,871 tumor samples, selected from a retrospective database, underwent comprehensive genomic profile. 6350 of the patients whose tumors were evaluated were male (53.5%) and 5,521 (46.5%) were female. Median age of the population was 60.2 years. Analyzed specimens were divided as follows: 5862 primary tumors, 403 lymph node metastases and 5606 distant organ metastases (the most frequent metastases coming from hepatic (*N* = 2334), peritoneal (*N* = 788) and pulmonary (*N* = 761) locations) [Fig. 1].

### Molecular differences between lymph node and distant organs metastases and colorectal primary tumors

**Fig. 2:**
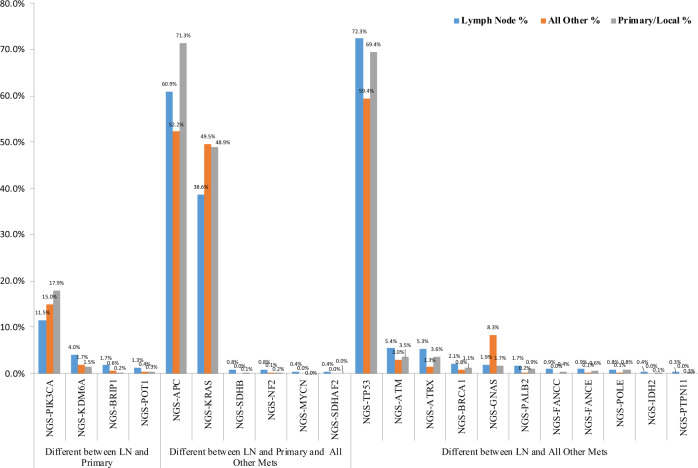
Molecular differences between tumor sites. Genes with significantly different mutation frequencies between lymph nodes (LN), distant metastases (mets) and colorectal primary tumors.

In this population, lymph node metastases exhibit distinct frequency patterns of gene mutations when compared to patterns observed in samples collected from primary tumors. Compared to primary tumors, significant higher frequencies of mutations in *KDM6A* (4% vs 1.5%), *BRIP1* (1.7% vs 0.2%) and *POT1* (1.3% vs 0.3%) genes were observed, while *PIK3CA* was less frequently mutated (11.5% vs 17.9%). Compared to all distant organ metastases, a higher frequency of mutations in lymph node metastases were noted in *TP53* (72.3% vs 59.4%), *ATM* (5.4% vs 3%), *ATRX* (5.3% vs 1.3%), *BRCA1* (2.1% vs 0.8%) and other genes such as *PALB2, FANCC, FANCE, POLE, IDH2* and *PTTPN11*. Only mutations in *GNAS* gene were more commonly found in distant organ metastases compared to lymph node metastases (1.9% vs 8.3%). Mutations in the *APC* gene were more frequent in primary tumors (71.3%), and a significant difference in prevalence was observed comparing the frequency in lymph node metastases and distant organ metastases (60.9% vs 52.2%). *KRAS* gene alterations were more common in primary (49.5%) and distant organ metastases (48.9%) when compared to lymph node metastases (38.6%) (Fig. 2).

### Immune-related biomarkers among lymph node and distant metastases and primary tumors

**Fig. 3:**
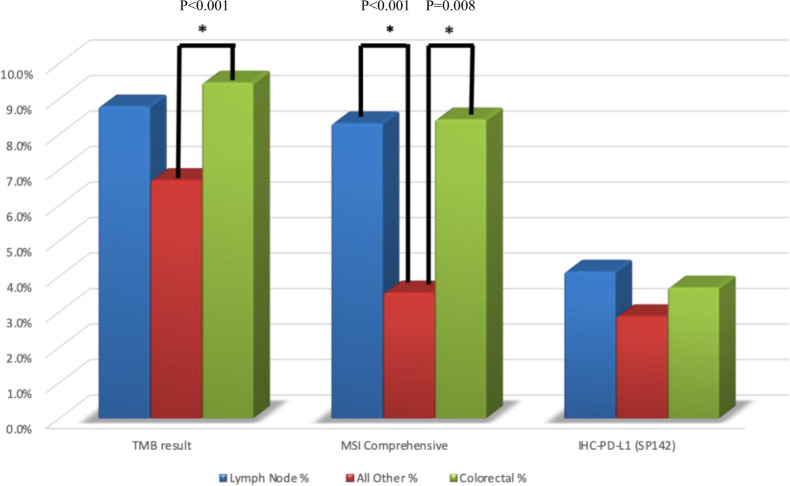
Difference in tumor mutational burden (TMB), microsatellite instability (MSI) and PD-L1 among lymph nodes, distant metastases, and primary colorectal tumors. Connective lines and asterisks indicate statistical significance.

**Fig. 4:**
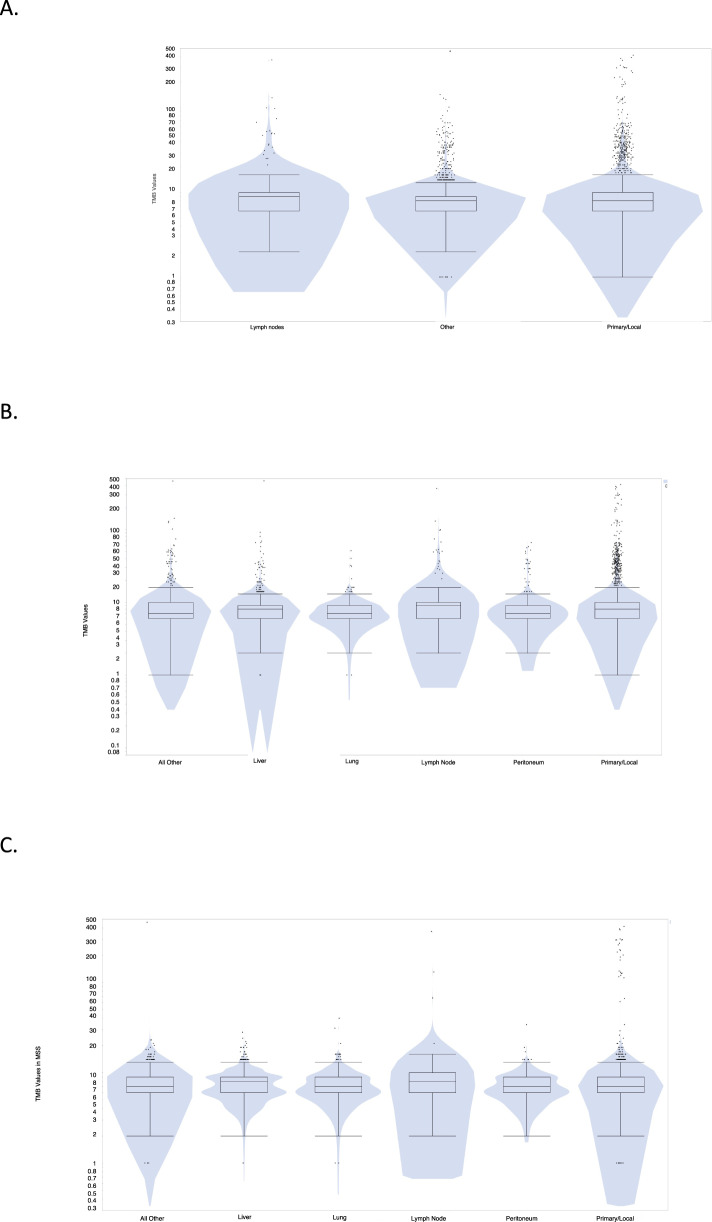
Tumor mutational burden (TMB) in lymph nodes (LNs), distant metastases and primary tumors in (b) all tumors and in (c) MSS only tumors. **A** Comparison of TMB in LNs (median TMB = 9), distant metastases (median TMB = 8) and colorectal primary tumors (median TMB = 8, *P* = 0.0052 by Kruskal–Wallis). **B** Comparison of TMBs with distant metastases further categorized into all distant metastases (median TMB = 7), liver (median TMB = 8), lung (median TMB = 7), LNs (median 17 TMB = 9), peritoneal (median TMB = 7), primary (median TMB = 8) (*P* = 0.0107 by Kruskal–Wallis). **C** Same categorization in microsatellite stable (MSS) tumors only: all distant metastases (median TMB = 7), liver (median TMB = 8), lung (median TMB = 7), LNs (median TMB = 8), peritoneal (median TMB = 7), primary (median TMB = 7) (*P* = 0.0001 by Kruskal–Wallis).

Overall, distant organ metastases show a lower frequency of TMB-high, MSI-H, and PD-L1 overexpression when compared to primary tumors and lymph node metastases which were noted to exhibit a similar prevalence of immune-related biomarkers. TMB-high was observed in 8.8% of lymph node metastases and 9.5% of primary tumors compared to 4.2% of distant organ metastases (*p* = 0.001 for lymph node metastases and *p* < 0.001 for primary vs distant metastases; no difference between primary and lymph node metastases, *p* = 0.73) (Fig. 3). The mean number of mutations per megabase (mt/Mb) in lymph node metastases is higher than in distant metastases (13.15 vs 9.11, *p* < 0.0001) and primary tumors (13.15 vs 11.66, *p* = 0.34). Among all metastatic sites, lymph node metastases are more frequently TMB-high. This characteristic is observed in all tumors, irrespective of MSI status (*p* < 0.001) and MSS tumor only (*p* = 0.027) (Fig. 4).

**Fig. 5:**
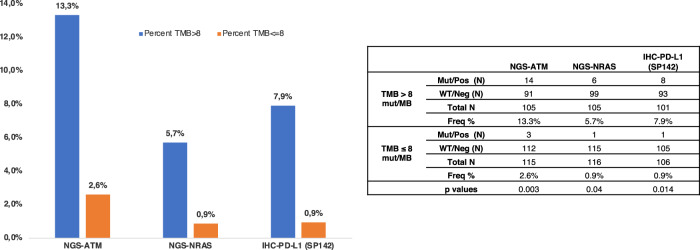
Molecular alterations associated with high tumor mutational burden (TMB) in microsatellite stable (MSS) lymph nodes metastases. MSS lymph nodes carrying TMB higher than median (i.e., 8 mut/MB) were compared to those that are lower, statistically significant results are shown.

Of note, TMB > 10 mt/MB was observed in 32% of lymph node metastases, in 24% of distant metastases, and in 25% in primary tumors. Next, MSS lymph node metastases were evaluated for differences in genetic alterations. Given the median number of mt/Mb, TMB-high lymph node metastases showed a higher frequency of *ATM* (13.3% vs 2.6%, *p* = 0.003) and *NRAS* (5.7% vs 0.9%, *p* = 0.04) mutations and more commonly manifested overexpression of PD-L1 (7.9% vs 0.9%, *p* = 0.014) [Fig. 5].

MSI-H status was present in 7.8% of lymph node metastases and 8.6% of primary tumors compared to 3.9% of distant organ metastases (*p* < 0.001 and *p* = 0.008 for lymph node metastases and primary vs distant organ metastases, respectively; no difference was found between primary and lymph node metastases, *p* = 0.64).

Finally, in all tumors, a higher frequency of PD-L1 overexpression was observed in primary tumors (3.7%) and lymph node metastases (4.1%) as compared to distant organ metastases (2.6%), however no statistical difference was observed (Fig. 3).

### Molecular profile of paired tumor samples

**Fig. 6:**
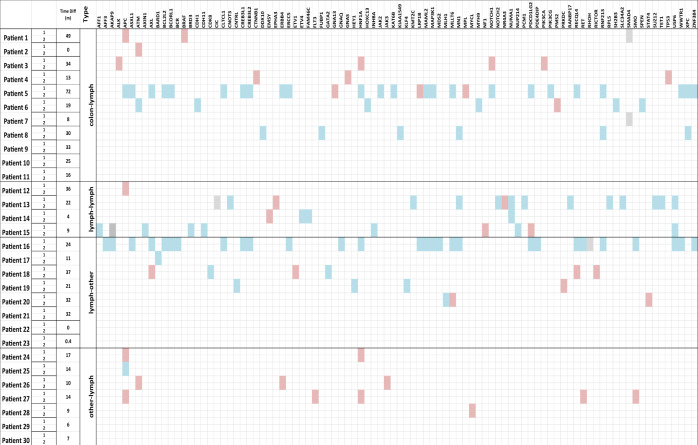
Oncoprint of paired samples. A total of 30 patients with lymph node samples were identified to have serial tumor samples profiled. 11 patients had one colon tumor and one lymph nodes sample profiled; 4 patients with two lymph nodes samples profiled; 15 tumors with one lymph nodes sample and one distant metastasis profiled. Red: acquisition (gain of function) of mutations; blue: loss of function mutations.

In an additional analysis, tumor specimens from thirty patients with sequentially obtained tissue samples taken from multiple sites were selected for paired sample profiling to identify molecular differences emerging over time (Fig. 6). Four different groups of paired samples were analyzed: colorectal primary and lymph node metastases samples (11 patients), two lymph node metastases samples (4 patients), lymph node metastases and distant organ metastases (8 patients) and distant metastatic sites and lymph node metastases samples (7 patients).

A gross total of 350 molecular alterations were observed: 34 pathogenic mutations, 22 variants of unknown significance (VUS), 121 alterations in wild-type genes, 57 unclassified mutations, 91 benign mutations, 2 presumed benign mutations and 16 indeterminate alterations. Among the pathogenic mutations the most commonly mutated genes were *APC* (*N* = 24), *SMAD4* (*N* = 3), *PIK3CA* (*N* = 2), *KRAS* (*N* = 2) and *NF1* (*N* = 2).

In the primary/lymph node metastases-paired group, 15 gain-of-function mutations were observed, two of them being pathogenic (1 in *APC* and 1 in *TP53*); 6 mutations were VUS of potentially pathogenic genes, such as *ALK, ATM, BRAF, PIK3CA*. Loss-of-function mutations were more frequent with 36 different gene alterations detected but observed in only three patients with one of them carrying 24 loss-of-function mutations between the two samples analyzed. In the lymph node metastases/lymph node metastases-paired samples group, 6 gain-of-function mutations were observed with only one being pathogenic (*APC*), 1 VUS (*NF1*) and 4 unclassified. Twenty-two loss-of-function mutations were identified, the great majority (12) in a single patient. Seven unclassified gain-of-function mutations were observed in the lymph node metastases/distant metastases-paired samples group. Thirty-five loss-of-function mutations were detected in 5 different patients; again, as for the colon/lymph node metastases and the lymph node metastases/lymph node metastases-group one single patient presented a high number of these alterations (25) while others showed a significantly smaller number (1).

In the distant metastases/lymph node metastases-paired samples group, 11 gain-of-function mutations were observed, two of them being pathogenic (*APC*), 2 VUS and 7 benign. Finally, for 8 patients no molecular alterations were observed between the samples analyzed.

## Discussion

Within this study we analyzed the genetic landscape of primary CRC tumors and compared the outcomes with the molecular profiles observed in lymph node and distant organ metastases. We observed that genetic alterations differ significantly between lymph node and distant organ metastases. Our study, therefore, challenges the “single-cell origin theory of metastasis”^14^ since the differences of the genetic landscape between lymph node and distant organ metastases suggests that, in contrast to this theory, a single clone is not responsible for tumor cell spreading. Rather tumor spread is more likely a consequence of multiple clones of tumor cells accounting for lymphatic and hematogenic tumor cell spreading. This is in line with another study conducted by Wei Q and colleagues. Using whole-exome sequencing they showed that the genetic landscape differs between primary tumors and their matched distant metastases^15^.

An attempt to refute the “single-cell origin theory of metastasis” was put forward by Nexerova et al.^4^ The authors examined the evolutionary relationship between primary CRC tumors, lymph node and distant metastases analyzing 20 to 43 noncoding regions that contain long, uninterrupted stretches of the nucleotide base guanine. These regions do not influence a cancer cell’s ability to metastasize, but their rapid mutation rate enabled the researchers to construct evolutionary trees for the primary tumors as well as distant and lymph node metastases^16^. In 65% of cases (11 out of 17), lymphatic and distant metastases arose from independent subclones from those observed in the primary tumor, whereas in 35% the tumor and the metastatic deposits shared a common subclonal origin. Interestingly, these findings were not influenced by common clinicopathological variables, e.g. treatment history. These results support the hypothesis that lymph nodes do not serve as a way station for the metastasizing cells, suggesting that spread to lymph nodes is not an essential intermediate step that predates distant metastatic spread, at least for CRC. The remaining 6 cases (35%) demonstrated that a common evolutionary branch connected distant organ metastasis to at least one lymph node deposit, which suggests that either cancer cells disseminated from nodes to distant organ sites, or that a dominant metastasis-competent clone in the primary tumor seeded, in parallel, both lymph nodes and distant organ sites^17^.

In this study, we found a higher frequency of *APC* mutations in primary tumors compared to lymph node and distant organ metastases. *APC* mutations activates WNT signaling that consequently up-regulates epithelial-to-mesenchymal transition (EMT)^18^. Activation of EMT leads to metastasis and reduced survival in patients with CRC^19^. As such, a lower *APC* mutation frequency in metastases and a higher rate in the primary tumor may be linked to EMT of tumor CRC cells. Furthermore, immune- related biomarkers differed significantly between lymph node and distant organ metastases and primary tumors. We have observed a high concordance of MSI-H between primary tumors and lymph node metastases. Interestingly, distant organ metastases harbor a significantly lower percentage rate of MSI-H tumors in comparison to primary tumors and lymph node metastases. This may be related to the better biology of the disease in MSI-H tumors, as supported also in previous studies^20^.

TMB-high status is significantly more common in lymph node compared to distant organ metastases and primary tumors. Of note, the difference remains significant also in the MSS subgroup. When comparing the molecular landscape of TMB-high lymph node metastases and other tumor locations, *ATM* genetic alterations were observed more frequently in the TMB-high lymph node metastases group. *ATM* belongs, like *BRCA*^21^ and *WRN*^22^, to the class of damage DNA repair (DDR) genes and recently published work showed that mutations in one of the DDR genes may be associated with a higher TMB^23^. As such, we hypothesize that the *ATM* mutation may be the trigger for higher TMB levels in MSS lymph node metastases.

We could hypothesize that the tumor cells with higher TMB within lymph nodes may harbor some genetic defects which might interfere with the metastatic process and thus less efficiently seed distant metastases. Further analyses are needed to clarify this hypothesis. Taken together, data coming from these studies are clearly showing that a single cancer cell is not solely responsible for tumor spreading but is more likely that a polyclonal process triggers tumor dissemination^24,25^. It is also clear that the mutational landscape of tumors evolves over time. This is particularly true in MSI-H tumors as noted by Le and colleagues^26^ who found that brain metastases in two patients had different mutational profiles from previously biopsied metastatic sites. Further studies, including single-cell sequencing techniques may be useful to discover what kind of mutations in tumor cells may facilitate lymphatic and hematological spreading. Furthermore, the impact of the intratumoral heterogeneity of CRC and tumor spreading as well as the tumor microenvironment including the gut microbiota should not be neglected as co-factors for tumor dissemination^27,28^. Variation seen in the genetic landscape in this study reflects the strong heterogeneity of CRC. Only recently, investigators deciphered the molecular profile of patients with right-sided and left-sided colon cancer primaries showing that there are several differences suggesting that right-sided tumors have distinctive profiles from left-sided tumors and have a worse prognosis^29,30^. Further studies should investigate whether tumor sidedness differentially impacts the genetic landscape of lymph node and distant organ metastases. Unfortunately, we were not able to answer this question since only limited clinico-pathological data were available in our database, thus we cannot correlate our findings with demographic or treatment related factors as well as survival data. Some further limitations exist in this study as well. The retrospective study design may have led to an inapparent selection bias. We acknowledge that the major limitation of the present study is that the vast majority of tumors analyzed were not matched and that the heterogeneity within the tumors was not analyzed. Larger datasets of matched samples with cancer evolution-focused analyses (i.e., compiled comparisons of primary, lymph node, and metastasis in each patient), are requested to properly discover relationships among primary, lymph node and distant CRC. From the data presented it is not possible to conclude whether the metastases arise from single clones or multiple clones due to positive and negative selection pressures.

Our data could support the hypothesis that each individual patient’s CRC is heterogeneous and evolves over time^31^, although further studies are needed to elucidate exactly how cancer cells metastasize and whether they arise from single clones or multiple clones within a heterogeneous evolving tumor.

This is one of the largest studies to investigate the molecular differences between lymph node and distant organ metastases and primary tumors in metastatic CRC patients. Lymph node metastases exhibit different genetic profiles compared to primary colorectal cancer and distant metastases. In addition, TMB-high, MSI-H and PD-L1 overexpression are more frequently seen in lymph nodes than in distant organ metastases, whereas in primary tumors a similar pattern is observed. Our findings are hypothesis generating and need to be examined in prospective studies.

## Methods

A total of 11,871 tumors that underwent comprehensive genomic profiling by a CLIA/CAP-certified laboratory (Caris Life Sciences; Phoenix, AZ) were identified from a retrospective database (molecular information were deidentified and stored in a biomarker database structured and organized based on cancer types and histological information). The tumors included in the study were consecutive colorectal tumors submitted to Caris Life Sciences by patients’ treating oncologists for tumor profiling tests in order to inform clinical decision.

Molecular characteristics, including microsatellite instability (MSI) status, tumor mutational burden (TMB), as well as protein expression by immunohistochemistry (IHC) were analyzed for differences based on tumor site: primary (*N* = 5862), distant organs metastases (*N* = 5606) and lymph nodes (*N* = 403).

### Immunohistochemistry

IHC was performed on formalin-fixed paraffin-embedded (FFPE) tumor sections. Protein staining was scored for intensity (0 = no staining; 1 += weak staining; 2 += moderate staining; 3 += strong staining) and staining percentage (0–100%) by pathologists. PD-L1 testing was performed using the anti-PD-L1 clone SP142 (Ventana, Tucson, AZ).

Immunohistochemistry (IHC) was performed on full formalin-fixed paraffin-embedded (FFPE) sections of glass slides. Slides were stained using automated staining techniques, per the manufacturer’s instructions, and were optimized and validated per CLIA/CAO and ISO requirements. Staining was scored for intensity (0 = no staining; 1 += weak staining; 2 += moderate staining; 3 += strong staining) and staining percentage (0–100%). Results were categorized as positive or negative by defined thresholds specific to each marker based on published clinical literature that associates biomarker status with patient responses to therapeutic agents. A board-certified pathologist evaluated all IHC results independently.

### Next-generation sequencing

NGS was performed on genomic DNA isolated from FFPE tumor samples using the NextSeq (592-genes) or MiSeq platform (47-gene) (Illumina, Inc., San Diego, CA). All variants were detected with greater than 99% confidence based on allele frequency and amplicon coverage, with an average sequencing depth of coverage of greater than 500 and an analytic sensitivity of 5%. Variants detected were mapped to reference genome (hg19) and well-established bioinformatics tools such as BWA, SamTools, GATK and snpFF were incorporated to perform variant calling functions; germline variants were filtered with various germline databases including 1000 genome and dbSNP. Matched normal tissue was not sequenced. A custom-designed SureSelect XT assay was used to enrich 592 whole-gene targets (Agilent Technologies, Santa Clara, CA) or 47 genes of interest using a modified Illumina TruSeq Amplicon Cancer panel. All variants were detected with >99% confidence based on allele frequency and amplicon coverage, with an average sequencing depth of coverage of >500 and an analytic sensitivity of 5%. Prior to molecular testing, tumor enrichment was achieved by harvesting targeted tissue using manual microdissection techniques. Genetic variants identified were interpreted by board-certified molecular geneticists and categorized as ‘pathogenic’, ‘presumed pathogenic’, ‘variant of unknown significance’, ‘presumed benign’, or ‘benign’, according to the American College of Medical Genetics and Genomics (ACMG) standards. When assessing mutation frequencies of individual genes’, pathogenic’, and ‘presumed pathogenic’ were counted as mutations while ‘benign’, ‘presumed benign’ variants and ‘variants of unknown significance’ were excluded.

### Microsatellite instability

MSI was examined by counting number of microsatellite loci that were altered by somatic insertion or deletion for each sample. The threshold to determine MSI by NGS was determined to be 46 or more loci with insertions or deletions to generate a sensitivity of >95% and specificity of >99%.

### Tumor mutational burden (TMB)

TMB was estimated from 592 genes (1.4 megabases [MB] sequenced per tumor) by counting all non-synonymous missense mutations found per tumor that had not been previously described as germline alterations.

### Statistical analysis

Differences in mean TMB were assessed using the ANOVA test. The Chi-square test was performed for comparative analysis using SPSS v23 (IBM SPSS Statistics), and significance was defined as *p* < 0.05. No special or customized scripts were used; all statistical analyses were performed using standard methods.

### Ethics statement

This study was conducted in accordance with guidelines of the Declaration of Helsinki, Belmont report, and U.S. Common rule. In keeping with 45 CFR 46.101(b)(4), this study was performed utilizing retrospective, deidentified clinical data. Therefore, this study was considered IRB exempt and no patient consent was required from the subjects.

### Reporting summary

Further information on research design is available in the Nature Research Reporting Summary linked to this article.

## Supplementary information

Reporting Summary

## Data Availability

The datasets generated during and/or analyzed during the current study are available from the corresponding author on reasonable request. The deidentified sequencing data are owned by Caris Life Sciences, and cannot be publicly shared due to the data usage agreement signed by Dr. Heinz Lenz. Qualified researchers can apply for access to these summarized data by contacting Joanne Xiu, PhD (jxiu@carisls.com) and signing a data usage agreement. The processed NGS sequencing data are available at https://figshare.com/articles/dataset/Supplemental_Data_xlsx/14854383.

## Code availability

No novel or customized scripts were used; all statistical analyses were performed using standard methods.
